# Clinical outcomes of patients with multiple courses of radiosurgery for brain metastases from non-small cell lung cancer

**DOI:** 10.1038/s41598-022-13853-3

**Published:** 2022-06-23

**Authors:** Won-Jae Lee, Jung-Won Choi, Doo-Sik Kong, Ho Jun Seol, Do-Hyun Nam, Jung-Il Lee

**Affiliations:** Department of Neurosurgery, Samsung Medical Center, Sungkyunkwan University School of Medicine, 81 Irwon-ro, Gangnam-gu, Seoul, 06351 South Korea

**Keywords:** CNS cancer, Oncology

## Abstract

We investigated the long-term clinical outcomes of patients who underwent multiple courses (≥ 5) of gamma knife radiosurgery (GKRS) due to recurrent brain metastases (BM) from non-small cell lung cancer (NSCLC). Between December 2001 and July 2019, consecutive 2571 patients underwent GKRS for BM from NSCLC. Clinical and radiological outcomes were investigated in 76 patients who underwent GKRS ≥ 5 times. The median follow-up period after the diagnosis of NSCLC was 54.6 months (range 14.5–159.1 months). The median number of GKRS procedures per patient was six (range 5–15). Actuarial post-GKRS survival rates at 1, 2, 3, 4, and 5 years following initial GKRS were 88.1%, 79.5%, 65.3%, 51.4%, and 37.3%, respectively. No significant difference in overall survival was observed between patients (*n* = 22) with whole-brain radiotherapy (WBRT) and patients (*n* = 54) without WBRT (*p* = 0.076). The incidence of radiation-induced leukoencephalopathy was 64% and 18% in patients with and without WBRT, respectively (*p* < 0.0001). Multiple courses of SRS are a tolerable and effective treatment option for recurrent BM from NSCLC. Repeat SRS may be an alternative treatment option to avoid or delay WBRT.

## Introduction

The lungs are the most common primary site of malignancy, resulting in brain metastases (BM)^1^ and approximately 30–50% of lung cancer patients develop BM^2^. Stereotactic radiosurgery (SRS), such as gamma knife radiosurgery (GKRS), is a well-established treatment option for BM. Based on the evidence established by multiple randomized trials, SRS has been used as a primary treatment for patients with limited (< 4) BM^3–6^. Although whole-brain radiotherapy (WBRT) has been shown to be greater distant control of BM than SRS alone, there was no significant benefit to the overall survival (OS)^7–10^. Further, the negative impact of WBRT on the cognitive functions of the patients makes the use of WBRT a matter of debate, especially for patients with good performance status^5,6,11^. Recently, the number of BM is no longer considered as a limiting factor for SRS^10,12–15^. Instead, many studies revealed that the tumor volume plays a role in the prognosis of BM after SRS^16–19^. With the advancement of systemic therapy improving the survival of cancer patients, the incidence of BM is increasing because the patients have a longer time window for cancer dissemination^20,21^. For those long-term survivors, the brain remains a site for continued distant relapse^22–24^. However, there is no generalized consensus regarding the optimal re-treatment option for patients with recurrent BM^25^. Although many centers currently adopted repeated SRS as a salvage treatment for local and distant recurrent BM, only limited literature reported their experience about multiple courses of SRS and long-term clinical information is lacking^24,26–28^.

In the present study, we investigated the long-term clinical outcomes of patients who underwent multiple courses (≥ 5 times) of GKRS due to recurrent BM from non-small cell lung cancer (NSCLC) to evaluate the efficacy and feasibility of multiple repeat GKRS.

## Materials and methods

### Study population

**Figure 1:**
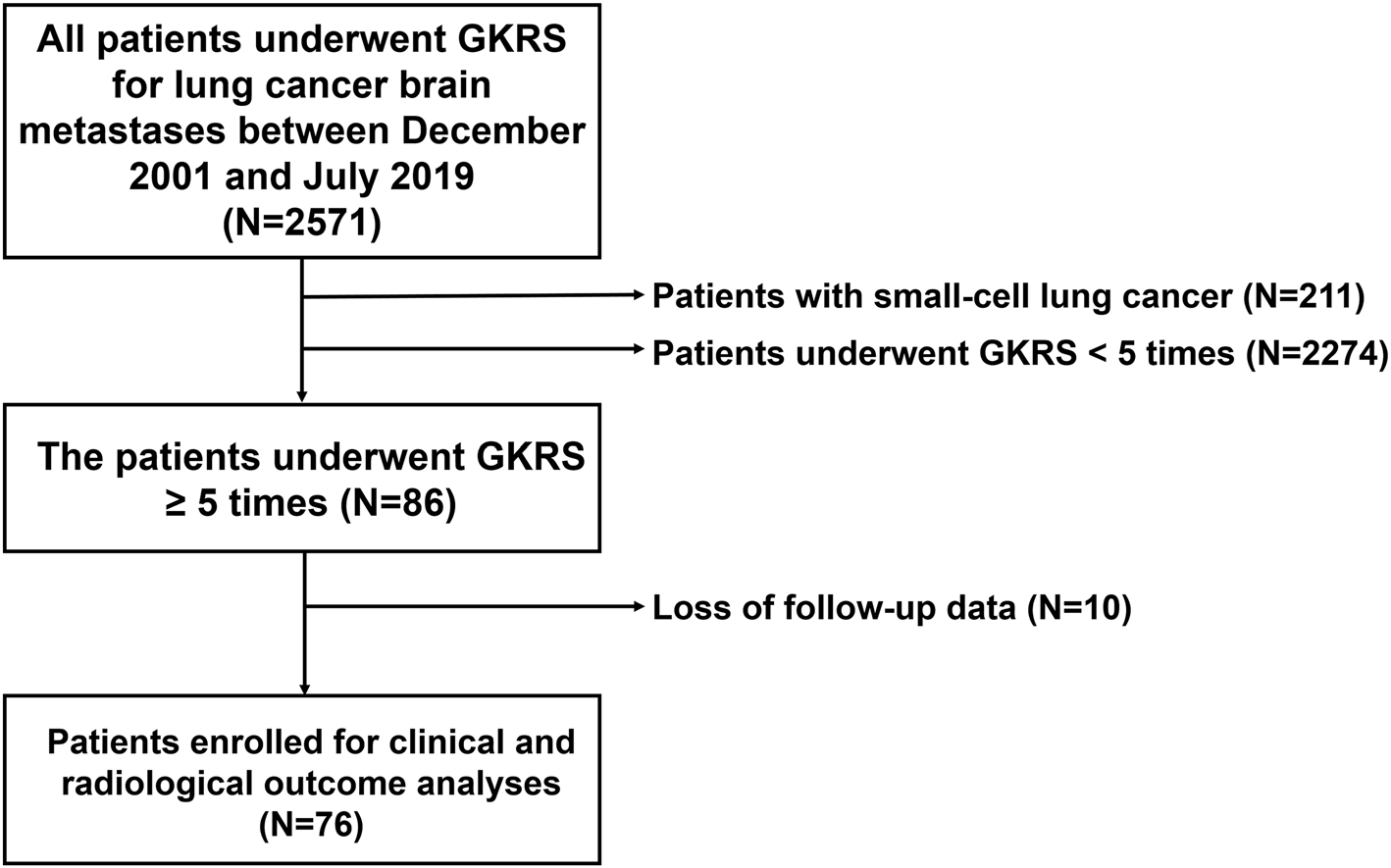
Flowchart showing the numbers of included and excluded patients.

This retrospective study was approved by the Institutional Review Board (IRB) of our institute (Samsung Medical Center Institutional Review Board [IRB number: 2020-02-062]) and conducted according to the ethical guidelines of the Declaration of Helsinki. The requirement for informed consent was waived off. Between December 2001 and July 2019, a total of 2571 consecutive patients underwent GKRS for BM from lung cancer in our center. The patients were stratified based on the total number of GKRS procedures they underwent; clinical and radiological outcomes were investigated in 76 patients with NSCLC who underwent GKRS ≥ 5 times (Fig. 1).

### Radiosurgery technique

SRS was performed using Leksell Gamma Knife type B, type C, Perfection, and Icon (Elekta AB, Stockholm, Sweden). According to the institutional standard protocol, as described previously, contrast-enhanced T1-weighted images with a slice thickness of 1.0 mm and T2-weighted fluid-attenuated inversion recovery (FLAIR) images with a slice thickness of 2.0 mm were acquired^29,30^. Target volume (TV) was defined as the contrast-enhanced volume of the tumor on T1-weighted post-contrast magnetic resonance images (MRI) scan without a margin of the surrounding brain tissue^31^. The tumor margin was delineated on each MRI scan to calculate the TV using the Gamma Plan software (Elekta AB, Stockholm, Sweden). Treatment planning, including the dose prescription, was performed by the neurosurgeons. Based on the institutional own practice, the prescription dose to the tumor margin was selected considering multiple factors: TV, location of the lesions, beam-on time, patient’s medical condition, and previous history of WBRT. Generally, maximum tolerable dose for each GKRS procedure was determined according to the guidelines of Radiation Therapy Oncology Group (RTOG) protocol 90–05, published by Shaw et al.^31^. The prescription dose was adjusted on a case-by-case basis, if necessary. The isodose line (IDL) was adjusted to acceptable tumor coverage. The most common selection was a 50% IDL. For large cystic lesions, Ommaya reservoir placement was performed to reduce the TV, before the GKRS^32^.

The fractionated treatment scheme (counted as a single procedure in this study) was selected for a patient who had a large tumor (> 40 mm) or a tumor adjacent to the eloquent area or cranial nerves at risk. Adaptive planning was performed if necessary^30^.

If a patient with numerous (> 10) brain metastases (BM) could not endure a long treatment time, a two-stage treatment scheme (counted as two procedures in this study) was chosen, as described previously^29^. Briefly, numerous lesions were treated separately on 2 days. The time interval between each procedure of the two-stage treatment scheme was approximately 2 weeks. Large lesions in the eloquent area were of higher priority. The beam-on time for each procedure was usually limited to 120 min. All lesions, untreated in the first session or newly appearing were treated in the second procedure. The time limit of this treatment strategy based on our center’s own experience was not a strict policy. If the patient was expected to be able to endure long treatment time, all lesions were treated in a single session. The main purpose of the two-staged treatment was to avoid WBRT by cover every lesion with minimal normal brain radiation. To investigate the cumulative effect of repeat GKRS regarding the radiation induced complications, the two-staged treatment was counted as two separate procedures in this study.

The patients treated with GKRS were followed up with MRI at 2-month intervals, and the cumulative radiological response after each GKRS procedure was investigated. Systemic therapy was conducted according to the judgment of the medical oncologist based on the patients’ conditions. If there were distant new lesions (distant failure) or regrowth of the previously irradiated lesion (local failure), multidisciplinary approaches such as surgery, WBRT, repeat GKRS, or a combined approach was taken into consideration based on the patients’ conditions. Local failure was defined as a histologically proven recurrence or a 25% increase in the area of enhancement with corresponding increased perfusion on perfusion-weighted MRI^33^.

**Figure 2:**
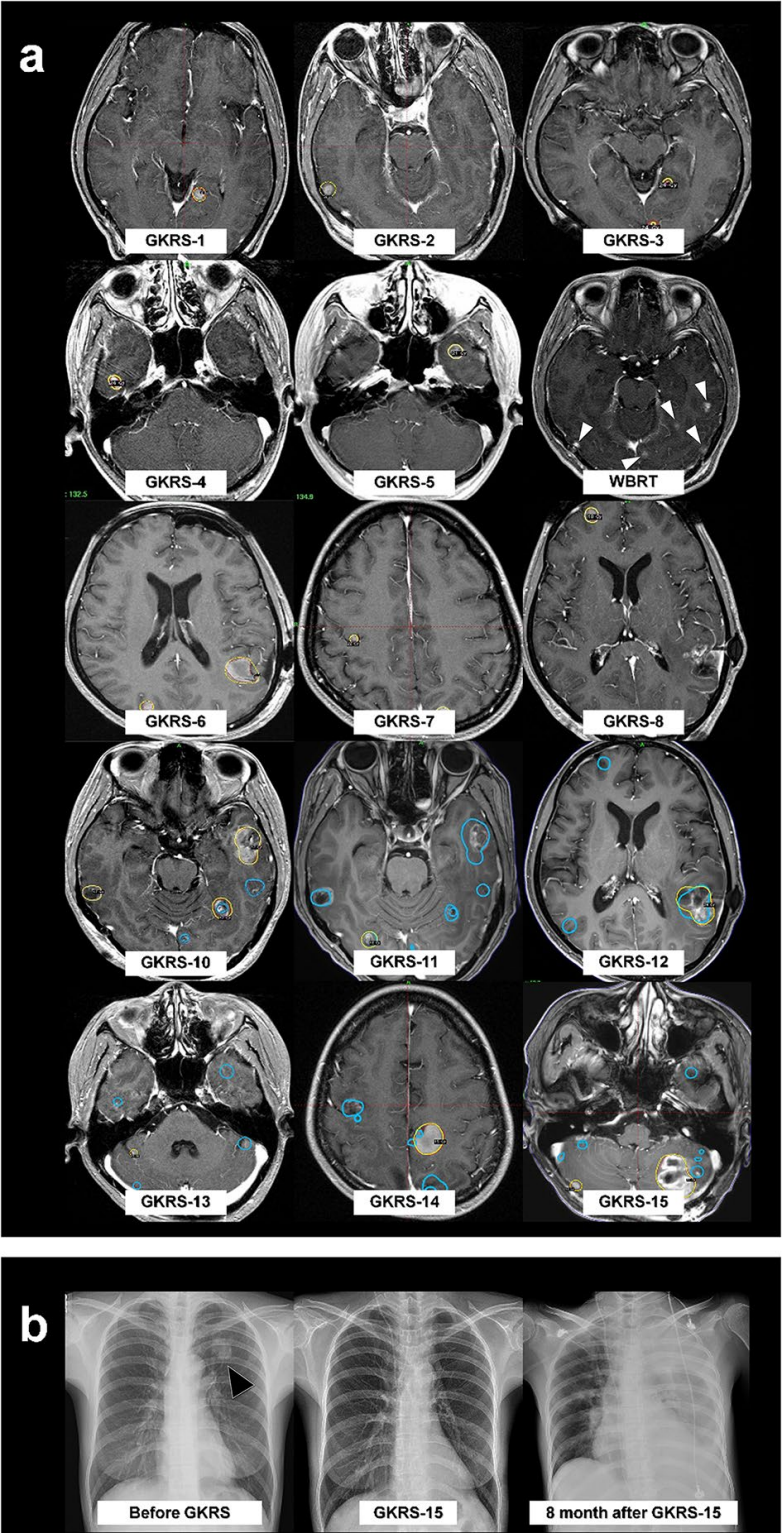
The clinical course of the a 36-year-old woman experiencing 15 GKRS procedures for BM from lung cancer. The patient was diagnosed with synchronous BM with adenocarcinoma of the lung. (**a**) Contrast-enhanced T1-weighted MR images for GKRS treatment. Target volume (red solid line) and prescription isodose volume (PIV) (yellow solid line) have appeared on each treatment planning image. PIVs of previously irradiated BM by gamma knife is marked with blue solid lines. The numerous lesions are indicated with white arrowheads resulting in an additional WBRT after the fifth GKRS. An Ommaya reservoir is inserted into a large cystic BM at the sixth GKRS. The newly developed hemorrhagic BM is shown in the cerebellum of the patient, resulting in metastasectomy and ventriculo-peritoneal shunt surgery after the 14th GKRS. (**b**) The chest X-rays showing lung cancer (black arrowhead) without systemic progression until the 15th GKRS (114 months after the first GKRS). Chest X-ray showing a large amount of pulmonary effusion on the left side after her death, following acute respiratory failure due to septic shock subsequent to 8 months of the last GKRS. *Data from the 9th GKRS session are lost.

An illustrative case of our treatment strategy was shown in Fig. 2. Repeat GKRS was a primary treatment modality for the patient with consecutive relapse of BM despite of multidisciplinary treatment. The use of WBRT was determined as a poor medical condition that could not endure the surgery or radiosurgical procedure, diffuse leptomeningeal dissemination, and rapidly progressive and miliary BM, which overwhelmed the treatment capacity of the two-stage treatment scheme.

### Outcomes assessment

Demographic data, clinical and radiological findings, parameters of GKRS, OS, and GKRS-related morbidities were reviewed. The cumulative number of targets for each patient was defined as the sum of the number of lesions treated at each GKRS procedure. Similarly, the cumulative prescription isodose volume (PIV) for each patient was defined as the summation of the PIV at each GKRS procedure. The median prescription dose for each patient during the course of GKRS treatment was investigated. If the patient had a heterogeneous prescription dose profile in a single procedure due to multiple lesions, the maximal value was used as the prescription dose for that procedure. For patients who underwent fractionated GKRS, the cumulative marginal dose was considered as the prescription dose of the procedure. The median value of the median prescription dose was investigated in each patient group stratified, based on the number of GKRS procedures they underwent.

**Figure 3:**
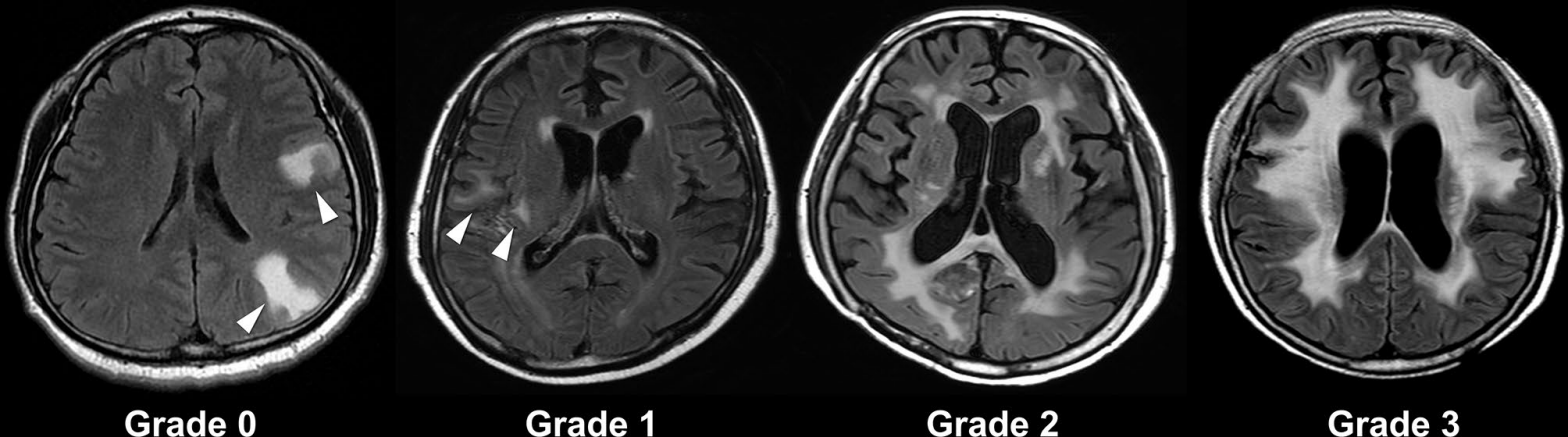
Fluid attenuation inversion recovery (FLAIR) images showing the white matter signal change. Radiation-induced leukoenchepalopathy (RIL) is classified into four grades using the grading system^34^. Tumor-related edema is not regarded as the RIL (white arrowheads).

The presence of radiation-induced leukoencephalopathy (RIL) was investigated in all patients and classified into four grades using the cerebral white matter (WM) change grading system developed by Fazekas et al.^34^. Figure 3 shows the newly developed hyperintense signal abnormalities surrounding the ventricles and the deep white matter (DWM) on FLAIR sequence after brain irradiation (0 = absence, 1 = “caps” or pencil-thin lining, 2 = smooth “halo”, 3 = irregular periventricular hyperintensity (PVH) extending into the DWM). Pre-irradiation WM signal changes, such as aging-related changes^35^ or peritumoral edema, were not regarded as RIL.

The cause of death in uncensored observations was investigated in two categories: neurological death and systemic disease progression. Neurological death was defined as death caused by progressive neurologic dysfunction due to uncontrolled BM. Patients with both intra- and extracranial tumor progression were included in neurological death because they indicated the treatment failure of repeat GKRS. Patients were considered to have died of systemic disease progression if they died due to progressive vital organ failure without neurologic dysfunction^36^.

The Karnofsky Performance Status (KPS) score was used to evaluate the ability of patients to maintain their health-related quality of life (HRQoL)^37^.

Radiation induced morbidities were categorized using the toxicity grading system of the RTOG and the European Organization for Research and Treatment of Cancer (EORTC)^38^.

### Statistical analysis

Data are presented as the median or mean and range for continuous variables, and as frequency and percentages for categorical variables. Median values and frequency comparisons were performed using Student’s t-test, chi-square (χ^2^) test, Fisher’s exact test, or Mann–Whitney U test, as appropriate. Univariate statistical analyses (logistic regression) were performed to assess the categorical and continuous variables. Variables with *p* < 0.1, in univariate analysis, were selected for multivariate models. The follow-up time and time-to-event outcomes were calculated from the date of the first GKRS to the date of the last follow-up or to the event of interest (e.g., death). The standard Kaplan–Meier method was used to estimate the OS. Statistical analyses were performed using SPSS Statistics, version 25 software (IBM Corporation, Armonk, New York, USA). Statistical significance was set at *p* < 0.05.

### Conference presentation

Part of this work was presented at “The 39th Annual Meeting of the Korean Neurosurgical Society” on 1 May 2021.

### Ethical approval

All procedures performed in studies involving human participants were in accordance with the ethical standards of the institutional and/or national research committee (Samsung Medical Center Institutional Review Board) and with the 1964 Helsinki declaration and its later amendments or comparable ethical standards. For this type of study formal consent is not required.

## Results

The characteristics of the 76 patients who underwent multiple courses (≥ 5) of the GKRS are described in Table 1. A total of 481 GKRS procedures were conducted, and the median number of GKRS procedures per patient was six (range 5–15). The median time interval between each GKRS procedure was 4.6 months (range 0.2–64.9 months).

**Table 1:** Characteristics of the 76 patients who underwent multiple courses (≥ 5) of GKRS.

Factors	Value
No. of patient (%)	76 (100)
Median age (range), years	57 (30–75)
Female:male	37:39
Median follow-up time from the diagnosis of NSCLC (range), months	54.6 (14.5–159.1)
Median follow-up time from the first GKRS (range), months	39.5 (12.3–136.0)
**Pathology (%)**	
Adenocarcinoma	76 (100)
Total no. of GKRS procedures	481
Median no. of GKRS procedure per patient (range)	6 (5–15)
Median cumulative no. of target per patient (range)	29.5 (5–103)
Median cumulative PIV per patient (range), cm^3^	17.2 (2.0–93.8)
Median time interval between each session (range), months	4.6 (0.2–64.9)
**WBRT (%)**	
Before the first GKRS	8 (11)
After the first GKRS	14 (18)
**Metastasectomy (%)**	
Before the first GKRS	3 (4)
After the first GKRS	14 (18)
No. of patient with oncogene mutation (%)	47 (62)
Targeted therapy (%)	41 (54)
**Frequency of mutated oncogene (%)**	
EGFR	32 (42)
ALK	13 (17)
BRAF	2 (3)
KRAS	2 (3)
Immunotherapy (%)	13 (17)
Intrathecal chemotherapy (%)	3 (4)
Hydrocephalus (%)	9 (12)
**Cumulative radiological responses after each GKRS procedure (%), n = 405**^a^	
Distant failure	280 (69)
Local failure	44 (11)
Both	81 (20)
**KPS score at first GKRS (%)**	
< 70	1 (1)
≥ 70	75 (99)
**KPS score at last GKRS (%)**	
< 70	12 (16)
≥ 70	64 (84)
**Changes of KPS score**	
Improved	8 (11)
No change	23 (30)
Worsened	45 (59)

*GKRS* gamma knife radiosurgery, *PIV* prescription isodose volume, *WBRT* whole-brain radiotherapy, *KPS* Karnofsky Performance status.

^a^Cumulative radiological responses were investigated in 405 cases of repeated GKRS procedures.

Before the first GKRS procedure, eight patients (11%) underwent WBRT. The cumulative radiation dose of WBRT ranged from 20 to 30 Gy. The use of WBRT was determined due to miliary BM in five patients, and diffuse leptomeningeal dissemination in three patients. Three (4%) patients underwent pre-GKRS metastasectomy and subsequently underwent the first GKRS at the tumor resection bed.

During the follow-up period after the first GKRS procedure, 14 patients (18%) underwent WBRT as salvage treatment due to the rapid dissemination of BM. Intracerebral progression was observed in all 14 patients, and 3 of the 14 patients showed additional leptomeningeal dissemination. Further, 14 (18%) patients underwent salvage surgery; eight were pathologically proven to have tumor recurrence (one patient due to distant failure and seven patients due to local failure). The other six patients underwent surgery due to radiation-induced necrosis (RIN) of previously irradiated lesions, causing an increase in the intracranial pressure. The genetic alterations of oncogenes were observed in 47 (62%) patients and EGFR mutation was the most frequent. Forty-one (54%) patients with oncogene mutation underwent targeted therapy. In addition, another 12 (16%) also received various target agents regardless of the presence of oncogene mutations. Thirteen (17%) patients underwent immunotherapy. Three (4%) patients underwent intrathecal chemotherapy due to diffuse leptomeningeal dissemination of BM. Nine (12%) patients underwent ventriculoperitoneal shunt (VPS) surgery for hydrocephalus (HCP) which occurred due to progressive BM (e.g., diffuse leptomeningeal seeding) in eight of nine patients. The other patient had an obstructive HCP due to peritumoral edema which was caused by RIN of a cerebellar lesion.

Except for the first 76 procedures of GKRS, the cumulative radiological response after each GKRS was investigated in 405 repeat GKRS procedures. Distant failure was observed in 361 patients (89%) following GKRS procedures. At the time of first GKRS procedure, 75 (99%) patients had the KPS score ≥ 70. During the follow-up period, the KPS scores improved in 8 (11%) patients, worsened in 45 (59%) patients, and did not change in 23 (30%) patients. At the time of last GKRS procedure, 64 (84%) patients had the KPS score ≥ 70.

GKRS-related complications included 12 (16%) cases of RIN and one (1%) case of seizure. Six out of 12 RIN patients underwent surgical resection of the lesion, while the other six patients underwent steroid therapy. One patient had generalized seizures a day after GKRS and was treated with an anti-epileptic drug. Five (7%) patients experienced Grade ≥ 3 toxicity according to the RTOG/EORTC grading system (Table 2).

**Table 2:** GKRS-related complications.

Factors	Value
**Complication (%)**	
Radiation induced necrosis	12 (16)
Seizure	1 (1)
**Acute toxicity**^a^	
Grade 2	3
Grade 3	2
**Late toxicity**^a^	
Grade 1	4
Grade 2	1
Grade 3	3

^a^RTOG/EORTC toxicity grading system^9^.

The radiosurgical parameters of each patient group based on the total number of GKRS procedures are described in Table 3. The number of BM at each GKRS procedure was shown in Supplementary Fig. 1.

**Table 3:** Parameters of radiosurgery.

Total no. of GKRS procedure	No. of patients, n = 76 (%)	Median cumulative no. of target per patient (range)	Median cumulative PIV per patient (range), cm^3^	Median value of the median prescription dose (range), Gy	Median follow up time after the first GKRS (range), months
5	31 (41)	24 (5–69)	14.2 (2.0–41.3)	18.0 (12.0–25.0)	31.9 (12.3–136.0)
6	20 (26)	27.5 (8–59)	15.6 (3.9–50.9)	19.5 (16.0–25.0)	43.5 (13.1–134.8)
7	13 (17)	34 (7–63)	18.9 (6.4–93.8)	20 (13.0–24.0)	48.0 (22.6–96.3)
8	5 (7)	38 (13–76)	32.0 (24.3–59.8)	18.5 (14.5–20.0)	59.3 (22.6–96.3)
9	3 (4)	68 (39–86)	40.9 (22.2–44.6)	18 (14–19)	39.3 (31.0–40.6)
11	3 (4)	92 (60–103)	45.1 (27.5–47.6)	16 (15–18)	73.2 (39.6–109.8)
15^a^	1 (1)	70	45.3	20.5	113.9

*GKRS* gamma knife radiosurgery, *PIV* prescription isodose volume.

^a^Data from the 9th GKRS session were lost.

### The OS

**Figure 4:**
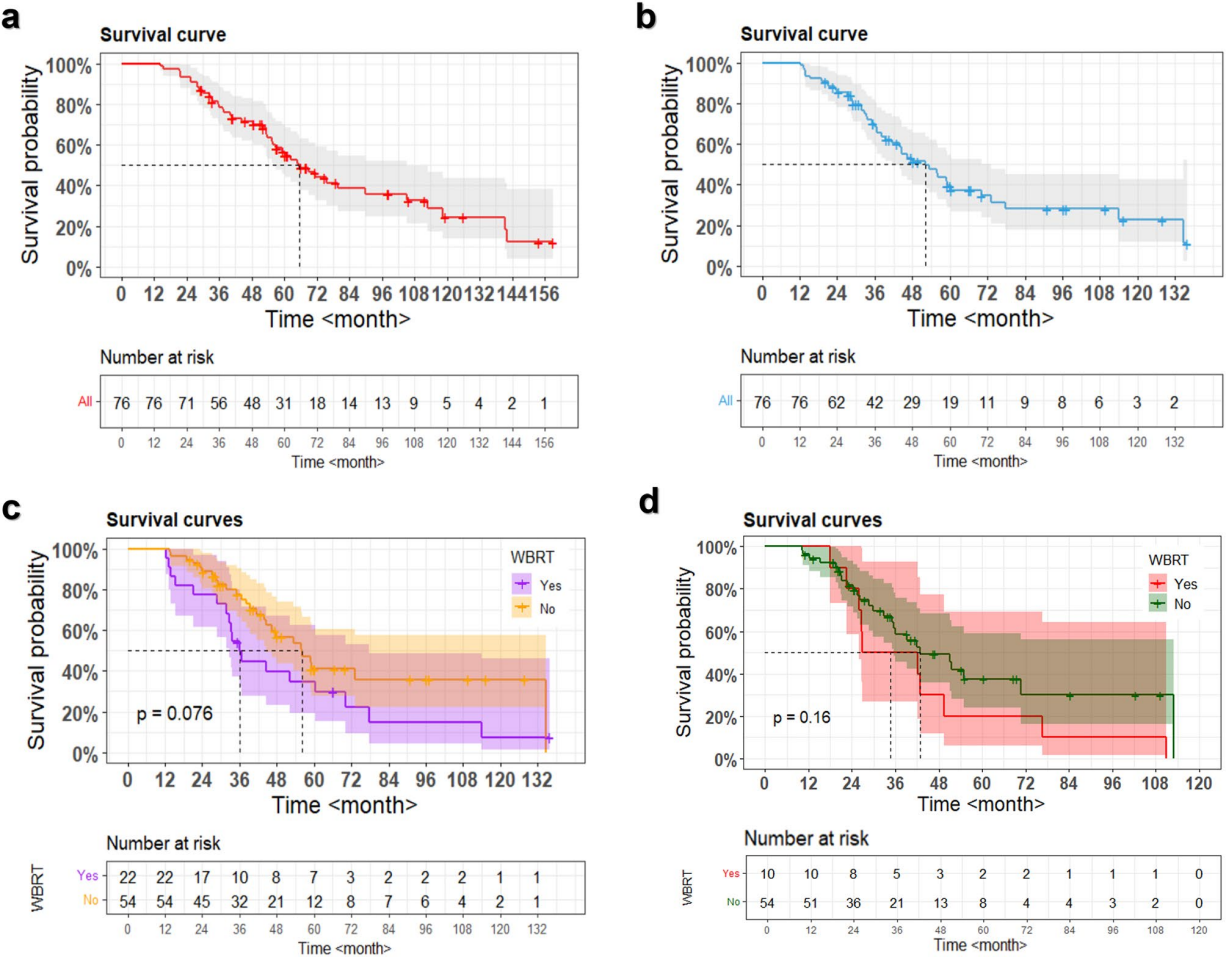
Kaplan–Meier survival curves. (**a**) A survival curve from the diagnosis of NSCLC. The median OS is 65.6 months (95% CI 53.42–77.78 months). (**b**) A survival curve from the first GKRS procedure. The median OS is 52.3 months (95% CI 41.47–63.13 months). (**c**) The median OS from the first GKRS procedure in patients with WBRT and the patients without WBRT is 35.9 months (95% CI 31.09–40.71 months) and 56.0 months (95% CI 42.34–69.66 months), respectively (*p* = 0.076). (**d**) The survival curves from the second GKRS in 64 patients without WBRT before the first GKRS. The median OS after the second GKRS in the 10 patients with salvage WBRT and the 54 patients without salvage WBRT was 27.0 months (95% CI 2.83–51.17) and 42.9 months (95% CI 28.97–56.83), respectively (p = 0.32). *The median OS is marked using a black dotted line in each figure. *OS* overall survival, *NSCLC* non-small cell lung cancer, *GKRS* gamma knife radiosurgery, *WBRT* whole-brain radiotherapy.

The median OS from the diagnosis of NSCLC was 65.6 months (95% confidence interval [CI] 53.42–77.78) (Fig. 4a). Actuarial survival rates at 1, 2, 3, 4, and 5 years were 86.8%, 73.0%, 66.7%, 54.8%, and 41.3%, respectively. The median OS from the first GKRS procedure was 52.3 months (95% CI 41.47–63.13) (Fig. 4b). The actuarial post-GKRS survival rates at 1, 2, 3, 4, and 5 years after the initial GKRS were 88.1%, 79.5%, 65.3%, 51.4%, and 37.3%, respectively.

In total, 56 patients died at the date of the closing of the study. Among the 44 uncensored observations, 37 (84%) patients died due to systemic disease progression and seven (16%) suffered a neurological death. As per the National Statistical Office database from Statistics Korea, death was confirmed for the rest 12 patients but the cause remained undetermined.

The median OS from the first GKRS procedure in patients with WBRT and patients without WBRT was 35.9 months (95% CI 31.09–40.71) and 56.0 months (95% CI 42.34–69.66), respectively (Fig. 4c). However, no significant difference in OS was observed (*p* = 0.076). There was no statistically significant difference in the baseline characteristics between the two groups, except for the patient’s age (Table 4).

**Table 4:** Comparison of the baseline characteristics of the two groups of patients according to the WBRT status.

Variables	WBRT		p-value	95% CI of the predicted differences
	Yes (n = 22)	No. (n = 54)		
Median age (range), years	57 (32–74)	60 (30–75)	0.001	− 14.09 to − 3.66
No. of females (%)	10 (45.5)	27 (50)	0.803	–
Median no. of GKRS procedure per patient (range)	6 (5–15)	6 (5–11)	0.462	− 1.24 to 0.57
Median cumulative no. of target (range)	29.5 (8–70)	28 (5–103)	0.494	− 7.07 to 14.52
Median cumulative PIV (range), cm^3^	17.2 (4.3–50.9)	16.9 (2.0–93.8)	0.974	− 8.29 to 8.57
Median value of median prescription dose (range), Gy	19 (12–24)	19 (13–25)	0.082	− 3.02 to 0.18

*GKRS *gamma knife radiosurgery, *PIV* prescription isodose volume, *WBRT* whole brain radiotherapy, *CI *confidence interval.

**Figure 5:**
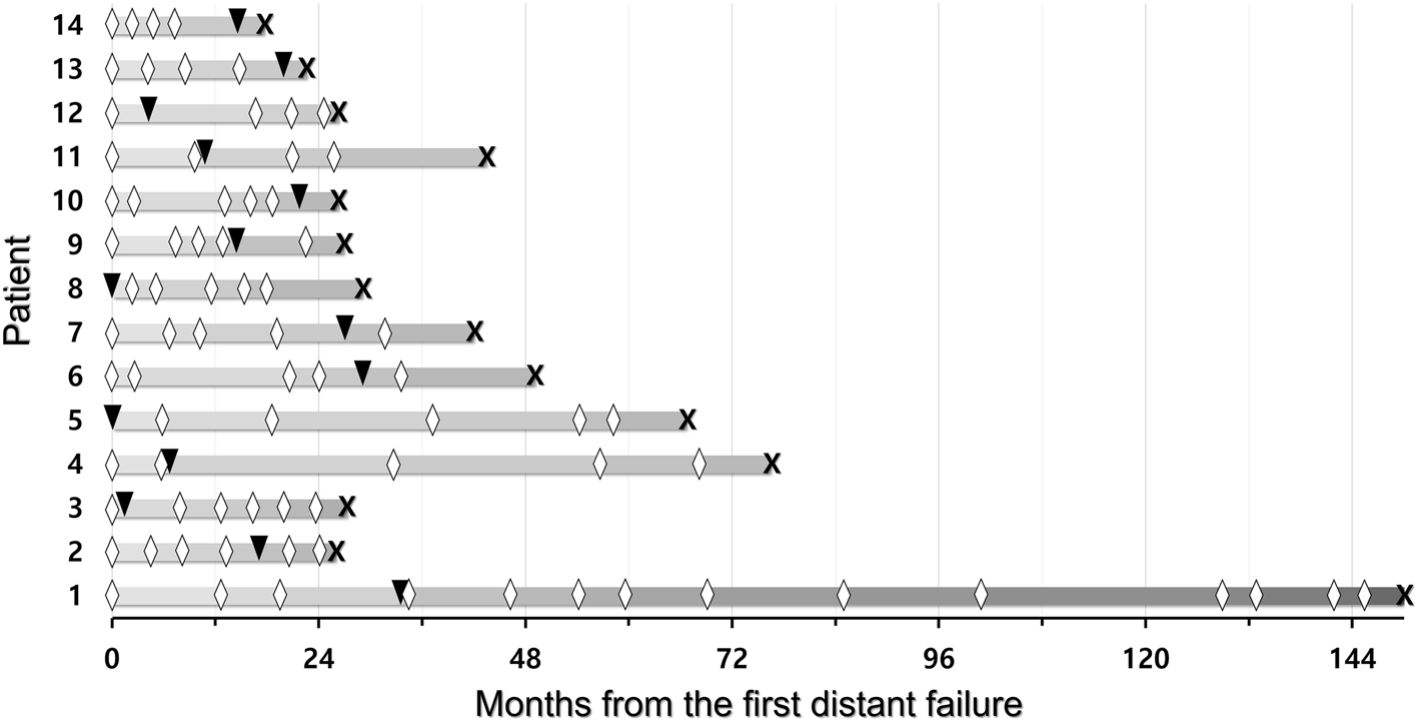
Timeline of the 14 patients who underwent salvage WBRT due to distant failure after the first GKRS. The diamond symbols indicate the repeat GKRS. The black arrow heads indicate the salvage WBRT. The cross “X” indicates the time of death. *GKRS* gamma knife radiosurgery, *WBRT* whole-brain radiotherapy.

In 68 patients without WBRT before the first GKRS, the median time interval from the first GKRS to the first distant failure was 7.3 months (range 1.0–89.2 months). Salvage WBRT was performed for the 14 patients at a various timing (Fig. 5). After the first distant failure, 2 patients underwent salvage WBRT, and the other 66 patients underwent second GKRS. The median OS after the second GKRS in the 10 patients with salvage WBRT and the 54 patients without salvage WBRT was 27.0 months (95% CI 2.83–51.17) and 42.9 months (95% CI 28.97–56.83), respectively (p = 0.32) (Fig. 4d). The actuarial survival rates of the 54 patients without salvage WBRT at 1, 2, 3, and 4 years after the second GKRS were 88.4%, 74.7%, 55.8%, and 37.5%, respectively.

### RIL cases

At the time of the first GKRS, a grade 1 RIL was observed in one of the eight patients who underwent WBRT before the first GKRS (Table 5). At the last follow-up, 24 (32%) patients had a RIL greater than grade 1. The incidence of RIL was 64% and 18% in patients with and without WBRT, respectively (*p* < 0.0001). The median time interval from the second GKRS to development of RIL in the 10 patients without WBRT was 23.1 months (range 9.0–89.8 months). The variables associated with RIL were analyzed separately for the all-patient group and the patient without WBRT group (Table 6). In the all-patient group, only WBRT was associated with RIL (*p* < 0.0001, odds ratio [OR] 7.70, 95% CI 2.55–25.30). In the group of patients without WBRT, age (*p* = 0.015, OR 1.15, 95% CI 1.03–1.29) and female sex (*p* = 0.05, OR 5.26, 95% CI 1.00–27.69) were found to be associated with RIL in univariate analyses. In multivariate analyses, only age (*p* = 0.04, OR 1.12, 95% CI 1.005–1.254) was found to be associated with RIL.

**Table 5:** Radiation-induced leukoencephalopathy.

Grade	All patients (*n* = 76)		WBRT			
			Yes (*n* = 22)		No (*n* = 54)	
	At the first GKRS (%)	At the last follow-up (%)	At the first GKRS (%)	At the last follow-up (%)	At the first GKRS (%)	At the last follow-up (%)
0	75 (99)	52 (68)	21 (95)	8 (36)	54 (100)	44 (82)
1	1 (1)^a^	12 (16)	1 (5)	5 (23)	–	7 (13)
2	–	2 (3)	–	1 (5)	–	1 (1)
3	–	10 (13)	–	8 (36)	–	2 (4)

*WBRT* whole brain radiotherapy, *GKRS* gamma knife radiosurgery.

^a^A grade 1 RIL was observed in one patient who underwent WBRT before the first GKRS.

**Table 6:** Factors associated with development of radiation-induced leukoencephalopathy based on univariate and multivariate analyses.

Variables	All-patient group (n = 76)	Patient group without WBRT (n = 54)	
	Univariate	Univariate	Multivariate
Age, years	0.49	0.015 (OR 1.15, 95% CI 1.03–1.29)	0.03 (OR 1.14, 95% CI 1.011–1.282)
Sex (female)	0.26	0.05 (OR 5.26, 95% CI 1.00–27.69)	0.15
WBRT	< 0.0001 (OR 7.70, 95% CI 2.55–23.30)	–	
No. of GKRS	0.35	0.43	
Cumulative no. of target	0.95	0.79	
Cumulative PIV, cm^3^	0.66	0.78	
Median prescription dose, Gy	0.18	0.68	

*GKRS* gamma knife radiosurgery, *PIV* prescription isodose volume, *WBRT* whole brain radiotherapy, *OR* Odds ratio, *CI* confidence interval.

## Discussion

This study showed the favorable salvage outcomes of the multiple courses of GKRS for the patients with recurrent BM from NSCLC. Repeat GKRS showed durable local control, and low neurological death (16%) was observed. Hence, it appears to be feasible salvage treatment option in the patients with recurrent BM. The role of SRS in the management of BM has expanded; in contrast, the use of WBRT is relatively decreasing^39^. Improvement in systemic therapies is allowing longer survival of cancer patients with BM, thereby preserving the cognitive functions, which is considered an important treatment goal for maintaining the HRQoL^37,40^. Several studies have revealed that SRS is superior in preserving the cognitive functions in patients with BM as compared to WBRT^5,6,11,41^. In the present study, a detailed assessment of the cognitive functions of the patients was not conducted. Neurocognitive decline was evaluated indirectly using the development of RILs and KPS scores due to retrospective nature. Although RILs have been roughly defined by cognitive dysfunction associated with diffuse WM change, clinical and neuropsychological investigation should be accompanied^42,43^. Nevertheless, the radiation-induced adverse effects due to WM integrity alteration have been well validated and the cognitive dysfunction is one of the most common and serious delayed complication of cerebral radiation^44–46^. Trifiletti et al. demonstrated that the addition of WBRT to SRS was associated with RIL^47^. Previous studies^47^ have indicated the association of the use of pre-SRS WBRT with an early RIL development. In the present study, WBRT was found to be associated with RIL, and patients with WBRT had a higher grade of RIL than patients without WBRT (Table 4). The incidence of RIL was significantly lower in patients without WBRT than in those with WBRT.

Increasing reports of cognitive decline following WBRT have resulted in increasing use of SRS as the sole treatment for BM^28^. A previously conducted prospective study (JLGK0901) reported that SRS without WBRT as the initial treatment in patients with multiple BM (5–10) is non-inferior to that of the patients with limited BM (2–4) in terms of OS^48^. The update of the JLGK0901 study focusing on cognitive functions and irradiation-related complications demonstrated the long-term safety of SRS alone in those with 5–10 BM^49^. Although SRS has no prophylactic effect for distant failure, this study showed that repeated SRS with short-term radiological follow-up could sufficiently overcome this concern. The survival outcome data also suggested that repeat SRS is a reasonable option for multiple recurrent BM. Patients with prolonged survival experienced a continuous relapse of BM (Table 2); however, additional WBRT had no impact on OS compared to SRS alone. The major cause of death in the uncensored observations was systemic disease progression, and repeated GKRS effectively controlled the intracranial burden of lung cancer. At the time of last GKRS procedure, 84% of the patients had the KPS score ≥ 70. Long-term self-care ability, measured by the KPS score, was relatively tolerable.

Over the past several decades, many centers have adopted SRS for patients with multiple (≥ 5) BM, and the number of BM itself was no longer a determinant for SRS eligibility^10,12,15,50^. Yamamoto et al. also demonstrated that the number of BM was not positively associated with SRS-related complications^12,50^. In the present study, SRS was performed as an initial or a salvage treatment for multiple BMs. For patients who underwent SRS as an initial treatment for BM, WBRT, which is generally considered to be unrepeatable, was reserved for miliary BM or leptomeningeal dissemination. In subgroup analysis, multiple BM developed in 67 (88%) patients, of which 45 (59%) patients could avoid the WBRT by repeating SRS (Supplementary Fig. 2). Although there are no randomized controlled trials evaluating the use of SRS alone in patients with multiple BM, SRS has moved into the mainstream treatment for multiple BM^51^. The results of our study encouraged the use of repeat SRS as a salvage treatment for multiple BMs to avoid radiation-induced brain damage. Multiple repeat SRS did not significantly increase the risk of RIL (Table 5). Recent technical advances in SRS have allowed a short treatment time for multiple lesions. The repeatability of SRS was a strong advantage in the management of recurrent distant failure. Repeat SRS at short intervals, such as a two-stage treatment scheme, can be considered as an alternative treatment methodology to replace WBRT. Given the HRQoL of patients, the decision to offer WBRT should be made on an individual basis. However, age was associated with RIL in patients who did not undergo WBRT. Physicians should be aware that elderly patients are vulnerable to radiation-induced cognitive decline, even if WBRT is omitted.

This study has several limitations. First, as a retrospective study, well-selected groups of patients might result in biased outcomes of multiple courses of GKRS. In addition, the effect of systemic therapy according to cancer biology, which could affect the prognosis of patients, was not evaluated. The regimens of chemotherapy, targeted therapy, and immunotherapy varied extremely among cases. Therefore, we were not able to use the systemic therapy as an independent variable. The survival outcome data may have time related bias that reflect the relatively superior outcome of systemic therapy in later years of the study. Future study considering the effect of systemic therapy will be needed to obtain more definitive results. The indications for initial or salvage WBRT should have been clearly defined. Moreover, cumulative radiation doses to the whole brain could not be determined due to technical limitations. Despite these limitations, this study reported long-term clinical outcomes of multiple courses of SRS in a single disease. Our results provide useful clinical information about the role of repeat SRS in patients with recurrent BM from NSCLC.

## Conclusions

Repeat SRS is a reasonable and effective treatment option for multiple recurrent BM from NSCLC. Additional WBRT increased the risk of RIL with no OS outcome. Short-term radiological follow-up is advocated in this treatment strategy to overcome the high risk of distant failure. The rate of symptomatic RIN was acceptable, and post-SRS HRQoL was tolerable. Multiple courses of SRS for recurrent BM may be an alternative treatment strategy to avoid or delay WBRT.

## Supplementary Information

Supplementary Figures.

## References

[1] Fox BD, Cheung VJ, Patel AJ, Suki D, Rao G (2011). Epidemiology of metastatic brain tumors. Neurosurg. Clin. N. Am..

[2] Sacks P, Rahman M (2020). Epidemiology of brain metastases. Neurosurg. Clin. N. Am..

[3] Sperduto PW (2014). Secondary analysis of RTOG 9508, a phase 3 randomized trial of whole-brain radiation therapy versus WBRT plus stereotactic radiosurgery in patients with 1–3 brain metastases; Poststratified by the graded prognostic assessment (GPA). Int. J. Radiat. Oncol. Biol. Phys..

[4] Andrews DW (2004). Whole brain radiation therapy with or without stereotactic radiosurgery boost for patients with one to three brain metastases: Phase III results of the RTOG 9508 randomised trial. Lancet (London, England).

[5] Aoyama H (2007). Neurocognitive function of patients with brain metastasis who received either whole brain radiotherapy plus stereotactic radiosurgery or radiosurgery alone. Int. J. Radiat. Oncol. Biol. Phys..

[6] Brown PD (2016). Effect of radiosurgery alone vs radiosurgery with whole brain radiation therapy on cognitive function in patients with 1 to 3 brain metastases: A randomized clinical trial. JAMA.

[7] Churilla TM (2017). Stereotactic radiosurgery with or without whole-brain radiation therapy for limited brain metastases: A secondary analysis of the north central cancer treatment group N0574 (alliance) randomized controlled trial. Int. J. Radiat. Oncol. Biol. Phys..

[8] Mulvenna P (2016). Dexamethasone and supportive care with or without whole brain radiotherapy in treating patients with non-small cell lung cancer with brain metastases unsuitable for resection or stereotactic radiotherapy (QUARTZ): Results from a phase 3, non-inferiority, randomised trial. Lancet (London, England).

[9] El Gantery MM, Abd El Baky HM, El Hossieny HA, Mahmoud M, Youssef O (2014). Management of brain metastases with stereotactic radiosurgery alone versus whole brain irradiation alone versus both. Radiat. Oncol..

[10] Sahgal A (2017). Stereotactic radiosurgery alone for multiple brain metastases? A review of clinical and technical issues. Neuro Oncol..

[11] Habets EJ (2016). Neurocognitive functioning and health-related quality of life in patients treated with stereotactic radiotherapy for brain metastases: A prospective study. Neuro Oncol..

[12] Yamamoto M (2014). Stereotactic radiosurgery for patients with multiple brain metastases: A case-matched study comparing treatment results for patients with 2–9 versus 10 or more tumors. J. Neurosurg..

[13] Serizawa T (2010). Gamma knife surgery for 1–10 brain metastases without prophylactic whole-brain radiation therapy: Analysis of cases meeting the Japanese prospective multi-institute study (JLGK0901) inclusion criteria. J. Neurooncol..

[14] Niranjan A, Monaco E, Flickinger J, Lunsford LD (2019). Guidelines for multiple brain metastases radiosurgery. Prog. Neurol. Surg..

[15] Hughes RT (2019). Initial SRS for patients with 5 to 15 brain metastases: Results of a multi-institutional experience. Int. J. Radiat. Oncol. Biol. Phys..

[16] Likhacheva A (2013). Predictors of survival in contemporary practice after initial radiosurgery for brain metastases. Int. J. Radiat. Oncol. Biol. Phys..

[17] Baschnagel AM (2013). Tumor volume as a predictor of survival and local control in patients with brain metastases treated with Gamma Knife surgery. J. Neurosurg..

[18] Hirshman BR (2018). Cumulative intracranial tumor volume augments the prognostic value of diagnosis-specific graded prognostic assessment model for survival in patients with melanoma cerebral metastases. Neurosurgery.

[19] Emery A (2017). More than just the number of brain metastases: Evaluating the impact of brain metastasis location and relative volume on overall survival after stereotactic radiosurgery. World Neurosurg..

[20] Moravan MJ (2020). Current multidisciplinary management of brain metastases. Cancer.

[21] Hochstenbag MM, Twijnstra A, Hofman P, Wouters EF, ten Velde GP (2003). MR-imaging of the brain of neurologic asymptomatic patients with large cell or adenocarcinoma of the lung. Does it influence prognosis and treatment?. Lung Cancer.

[22] Sundermeyer ML, Meropol NJ, Rogatko A, Wang H, Cohen SJ (2005). Changing patterns of bone and brain metastases in patients with colorectal cancer. Clin. Colorectal Cancer.

[23] Smith DR (2019). Natural history, clinical course and predictors of interval time from initial diagnosis to development of subsequent NSCLC brain metastases. J. Neurooncol..

[24] Kotecha R (2017). Three or more courses of stereotactic radiosurgery for patients with multiply recurrent brain metastases. Neurosurgery.

[25] Ammirati M (2010). The role of retreatment in the management of recurrent/progressive brain metastases: A systematic review and evidence-based clinical practice guideline. J. Neurooncol..

[26] Fritz C (2018). Repeated courses of radiosurgery for new brain metastases to defer whole brain radiotherapy: Feasibility and outcome with validation of the new prognostic metric brain metastasis velocity. Front. Oncol..

[27] Wowra B (2002). Repeated gamma knife surgery for multiple brain metastases from renal cell carcinoma. J. Neurosurg..

[28] Shen CJ (2016). The strategy of repeat stereotactic radiosurgery without whole brain radiation treatment for new brain metastases: Outcomes and implications for follow-up monitoring. Pract. Radiat. Oncol..

[29] Kim M (2020). Two-staged gamma knife radiosurgery for treatment of numerous (>10) brain metastases. Clin. Neurol. Neurosurg..

[30] Lee MH (2019). Volumetric changes of intracranial metastases during the course of fractionated stereotactic radiosurgery and significance of adaptive planning. J. Neurosurg..

[31] Shaw E (2000). Single dose radiosurgical treatment of recurrent previously irradiated primary brain tumors and brain metastases: Final report of RTOG protocol 90–05. Int. J. Radiat. Oncol. Biol. Phys..

[32] Yamanaka Y (2006). Ommaya reservoir placement followed by Gamma Knife surgery for large cystic metastatic brain tumors. J. Neurosurg..

[33] McKay WH (2017). Repeat stereotactic radiosurgery as salvage therapy for locally recurrent brain metastases previously treated with radiosurgery. J. Neurosurg..

[34] Fazekas F, Chawluk JB, Alavi A, Hurtig HI, Zimmerman RA (1987). MR signal abnormalities at 1.5 T in Alzheimer's dementia and normal aging. AJR Am. J. Roentgenol..

[35] Kim KW, MacFall JR, Payne ME (2008). Classification of white matter lesions on magnetic resonance imaging in elderly persons. Biol. Psychiatry.

[36] Patchell RA (1998). Postoperative radiotherapy in the treatment of single metastases to the brain: A randomized trial. JAMA.

[37] Bragstad S (2018). Predictors of quality of life and survival following Gamma Knife surgery for lung cancer brain metastases: A prospective study. J. Neurosurg..

[38] Cox JD, Stetz J, Pajak TF (1995). Toxicity criteria of the Radiation Therapy Oncology Group (RTOG) and the European Organization for Research and Treatment of Cancer (EORTC). Int. J. Radiat. Oncol. Biol. Phys..

[39] Ong WL, Wada M, Ruben J, Foroudi F, Millar J (2019). Contemporary practice patterns of stereotactic radiosurgery for brain metastasis: A review of published Australian literature. J. Med. Imaging Radiat. Oncol..

[40] Verhaak E (2021). Health-related quality of life after Gamma Knife radiosurgery in patients with 1–10 brain metastases. J. Cancer Res. Clin. Oncol..

[41] Chang EL (2009). Neurocognition in patients with brain metastases treated with radiosurgery or radiosurgery plus whole-brain irradiation: A randomised controlled trial. Lancet Oncol..

[42] Bompaire F (2018). New insights in radiation-induced leukoencephalopathy: A prospective cross-sectional study. Support Care Cancer.

[43] Zeng H (2020). Risk factors for neurocognitive decline in lung cancer patients treated with prophylactic cranial irradiation: A systematic review. Cancer Treat. Rev..

[44] Bargiotas I (2018). Balance impairment in radiation induced leukoencephalopathy patients is coupled with altered visual attention in natural tasks. Front. Neurol..

[45] Schuitema I (2013). Accelerated aging, decreased white matter integrity, and associated neuropsychological dysfunction 25 years after pediatric lymphoid malignancies. J. Clin. Oncol..

[46] Soussain C (2009). CNS complications of radiotherapy and chemotherapy. Lancet.

[47] Trifiletti DM (2015). Leukoencephalopathy after stereotactic radiosurgery for brain metastases. Int. J. Radiat. Oncol. Biol. Phys..

[48] Yamamoto M (2014). Stereotactic radiosurgery for patients with multiple brain metastases (JLGK0901): A multi-institutional prospective observational study. Lancet Oncol..

[49] Yamamoto M (2017). A multi-institutional prospective observational study of stereotactic radiosurgery for patients with multiple brain metastases (JLGK0901 Study Update): Irradiation-related complications and long-term maintenance of Mini-Mental State Examination Scores. Int. J. Radiat. Oncol. Biol. Phys..

[50] Yamamoto M (2013). A case-matched study of stereotactic radiosurgery for patients with multiple brain metastases: Comparing treatment results for 1–4 vs >/= 5 tumors: Clinical article. J. Neurosurg..

[51] Kraft J, Zindler J, Minniti G, Guckenberger M, Andratschke N (2019). Stereotactic radiosurgery for multiple brain metastases. Curr. Treat. Opt. Neurol..

