# Supplementary material for: Clinical outcomes of patients with multiple courses of radiosurgery for brain metastases from non-small cell lung cancer
Source: Sci Rep. 2022 Jun 23;12:10712. doi: 10.1038/s41598-022-13853-3 (PMC9226031; doi:10.1038/s41598-022-13853-3)
Supplement: Supplementary file 1 — Supplementary Figures. [file 41598_2022_13853_MOESM1_ESM.pdf]

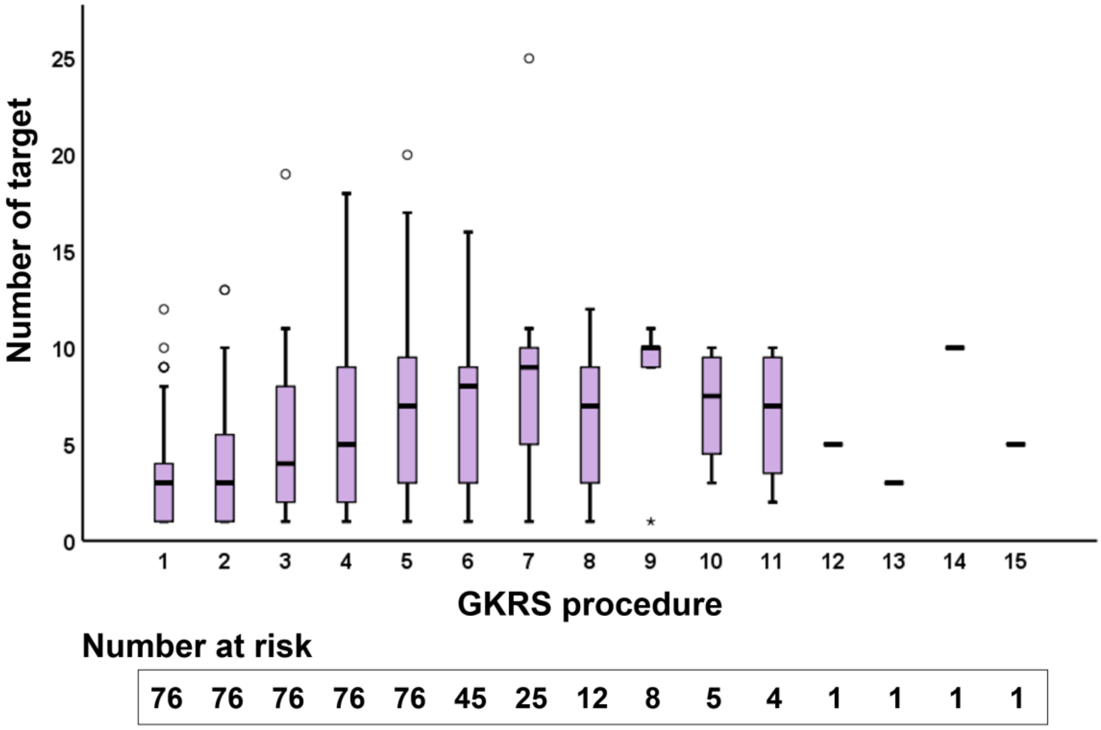

Supplementary Fig. 1 The box and whisker plots illustrate the number of BM at each GKRS procedure. The boxes indicate the 25th and 75th percentiles. The whisker indicates the minimum and maximum value, dots indicate the outliers, asterisk indicates extreme values, and thick horizontal lines indicate the median value.

BM, brain metastases; GKRS, gamma knife radiosurgery.

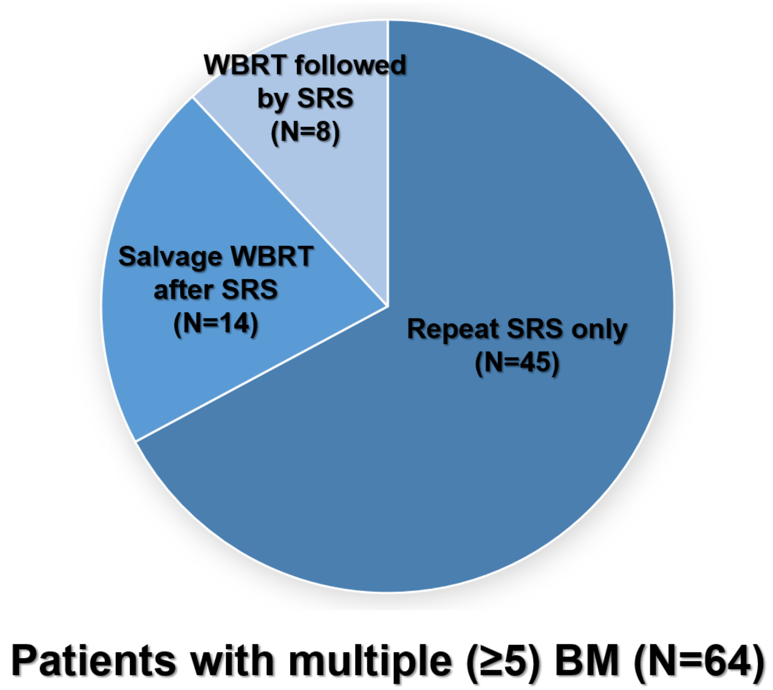

Supplementary Fig. 2 Treatment of patient with multiple (≥ 5) brain metastases (BM).

SRS, stereotactic radiosurgery; WBRT, whole-brain radiotherapy.
